# Supplementary material for: Association of Drinking Herbal Tea with Activities of Daily Living among Elderly: A Latent Class Analysis
Source: Nutrients. 2023 Jun 19;15(12):2796. doi: 10.3390/nu15122796 (PMC10305550; doi:10.3390/nu15122796)
Supplement: Supplementary file 1 [file nutrients-15-02796-s001.zip › nutrients-2446128-supplementary.pdf]

Supplementary Table S1. The probabilities of drinking herbal tea and tea in three-latent-class.

	Class 1	Class 2	Class 3
**Drinking herbal tea**			
**At aged 60 (2008 survey)**			
One almost every day	0.063	0.001	0.000
Two, not every day, but at least once per week	0.640	0.000	0.000
Three, not every week, but at least once per month	0.174	0.000	0.005
Four, not every month, but occasionally	0.123	0.989	0.054
Five rarely or never	0.000	0.010	0.941
**2008 survey**			
One almost every day	0.605	0.003	0.008
Two, not every day, but at least once per week	0.131	0.000	0.024
Three, not every week, but at least once per month	0.161	0.954	0.042
Four, not every month, but occasionally	0.104	0.042	0.079
Five rarely or never	0.000	0.001	0.847
**2011 survey**			
One almost every day	0.654	0.001	0.071
Two, not every day, but at least once per week	0.063	0.002	0.052
Three, not every week, but at least once per month	0.080	0.011	0.068
Four, not every month, but occasionally	0.151	0.928	0.166
Five rarely or never	0.053	0.058	0.643
**2014 survey**			
One almost every day	0.618	0.000	0.070
Two, not every day, but at least once per week	0.103	0.003	0.060
Three, not every week, but at least once per month	0.047	0.000	0.113
Four, not every month, but occasionally	0.173	0.961	0.200
Five rarely or never	0.058	0.036	0.557
**2018 survey**			
One almost every day	0.062	0.005	0.072
Two, not every day, but at least once per week	0.635	0.018	0.067
Three, not every week, but at least once per month	0.081	0.934	0.059
Four, not every month, but occasionally	0.147	0.032	0.140
Five rarely or never	0.075	0.010	0.663
**Drinking Tea**			
**At aged 60 (2008 survey)**			
One almost every day	0.730	0.087	0.001
Two, not every day, but at least once per week	0.095	0.155	0.013
Three, not every week, but at least once per month	0.015	0.703	0.012
Four, not every month, but occasionally	0.086	0.011	0.120
Five rarely or never	0.074	0.043	0.855
**2008 survey**			
One almost every day	0.802	0.144	0.000
Two, not every day, but at least once per week	0.066	0.059	0.012
Three, not every week, but at least once per month	0.019	0.778	0.014
Four, not every month, but occasionally	0.059	0.010	0.107
Five rarely or never	0.055	0.009	0.867

**2011 survey**			
One almost every day	0.091	0.211	0.105
Two, not every day, but at least once per week	0.757	0.086	0.052
Three, not every week, but at least once per month	0.008	0.037	0.035
Four, not every month, but occasionally	0.049	0.584	0.066
Five rarely or never	0.095	0.082	0.742
**2014 survey**			
One almost every day	0.732	0.069	0.073
Two, not every day, but at least once per week	0.102	0.089	0.037
Three, not every week, but at least once per month	0.021	0.744	0.028
Four, not every month, but occasionally	0.040	0.056	0.054
Five rarely or never	0.106	0.043	0.808
**2018 survey**			
One almost every day	0.100	0.041	0.049
Two, not every day, but at least once per week	0.589	0.040	0.034
Three, not every week, but at least once per month	0.028	0.008	0.011
Four, not every month, but occasionally	0.050	0.890	0.024
Five rarely or never	0.233	0.021	0.883

Note: Class 1 was defined as a frequently drinking group, Class 2 as an occasionally drinking group, and Class 3 as a rarely drinking group.

Supplementary Table S2. Baseline characteristics of the participants by different groups of drinking tea (N(%)).

Characteristics	N(%)	Frequently	Occasionally	Rarely	P-Value
Total	7441(100.0)	2203(29.6)	2095(28.2)	3143(42.2)	
**Sociodemographic characteristics**					
Age group, years					<0.001
<80	3209(43.1)	1012(31.5)	967(30.1)	1230(38.3)	
80-90	2309(31.0)	699(30.3)	675(29.2)	935(40.5)	
>=90	1923(25.8)	492(25.6)	453(23.6)	978(50.9)	
Gender					<0.001
Male	3485(46.8)	1321(37.9)	1030(29.6)	1134(32.5)	
Female	3956(53.2)	882(22.3)	1065(26.9)	2009(50.8)	
Education Level					<0.001
Illiteracy	4129(55.5)	991(24.0)	1131(27.4)	2007(48.6)	
Primary School or Below	1887(25.4)	673(35.7)	538(28.5)	676(35.8)	
Junior High School or Above	1425(19.2)	539(37.8)	426(29.9)	460(32.3)	
Ethnic					<0.001
Han	6964(93.6)	2132(30.6)	2028(29.1)	2804(40.3)	
Other	477(6.4)	71(14.9)	67(14.1)	339(71.1)	
Residence Area					<0.001
City	1178(15.8)	413(35.1)	336(28.5)	429(36.4)	
Town	1599(21.5)	575(36.0)	499(31.2)	525(32.8)	
Rural	4664(62.7)	1215(26.1)	1260(27.0)	2189(46.9)	

Co-Residence					0.012
With Household Member(S)	6058(81.4)	1834(30.3)	1708(28.2)	2516(41.5)	
Alone or In an Institution	1383(18.6)	369(26.7)	387(28.0)	627(45.3)	
**Healthy lifestyle and status**					
Smoking					<0.001
Never	4722(63.5)	1144(24.2)	1279(27.1)	2299(48.7)	
Current or Former	2719(36.5)	1059(39.0)	816(30.0)	844(31.0)	
Drinking					<0.001
Never	4941(66.4)	1275(25.8)	1316(26.6)	2350(47.6)	
Current or Former	2500(33.6)	928(37.1)	779(31.2)	793(31.7)	
Drinking herbal tea					<0.001
Frequently	2203(29.6)	344(38.4)	292(32.6)	260(29.0)	
Occasionally	2095(28.2)	712(37.3)	624(32.7)	575(30.1)	
Rarely	3143(42.2)	1147(24.8)	1179(25.4)	2308(49.8)	
Eat Fresh Fruits					<0.001
Almost Everyday	2895(38.9)	1006(34.8)	830(28.7)	1059(36.6)	
Occasionally	2807(37.7)	779(27.8)	787(28.0)	1241(44.2)	
Rarely or Never	1739(23.4)	418(24.0)	478(27.5)	843(48.5)	
Eat Fresh Vegetables					<0.001
Almost Everyday	6732(90.5)	2045(30.4)	1861(27.6)	2826(42.0)	
Occasionally or rarely	709(9.5)	158(22.3)	234(33.0)	317(44.7)	
Exercise					<0.001
Current	2588(34.8)	843(32.6)	731(28.3)	1014(39.2)	
Former	703(9.4)	239(34.0)	194(27.6)	270(38.4)	
Never	4150(55.8)	1121(27.0)	1170(28.2)	1859(44.8)	
Self-Reported Health					<0.001
Good or Very Good	3831(51.5)	1239(32.3)	1084(28.3)	1508(39.4)	
General or Below	3610(48.5)	964(26.7)	1011(28.0)	1635(45.3)	
No. of comorbidities					0.286
0	4822(64.8)	1397(29.0)	1357(28.1)	2068(42.9)	
1	1944(26.1)	614(31.6)	548(28.2)	782(40.2)	
2	530(7.1)	147(27.7)	149(28.1)	234(44.2)	
3	120(1.6)	34(28.3)	34(28.3)	52(43.3)	
⩾4	25(0.3)	11(44.0)	7(28.0)	7(28.0)	
